# Evaluation of Diagnostic Value in Using a Panel of Multiple Tumor-Associated Antigens for Immunodiagnosis of Cancer

**DOI:** 10.1155/2014/512540

**Published:** 2014-04-13

**Authors:** Peng Wang, Chunhua Song, Weihong Xie, Hua Ye, Kaijuan Wang, Liping Dai, Yi Zhang, Jianying Zhang

**Affiliations:** Center for Tumor Biotherapy, The First Affiliated Hospital and College of Public Health and Henan Key Laboratory of Tumor Epidemiology, Zhengzhou University, Zhengzhou, Henan 450052, China

## Abstract

To determine whether a panel of multiple tumor-associated antigens (TAAs) would enhance antibody detection, the diagnostic value of autoantibodies to a panel of multiple TAAs in cancer has been evaluated. The TAAs used in this study was composed of eight TAAs including Imp1, p62, Koc, p53, C-myc, Cyclin B1, Survivin, and p16 full-length recombinant proteins. Enzyme-linked immunosorbent assay and immunoblotting were used to detect antibodies in 304 cancer sera and also 58 sera from normal individuals. The antibody frequency to any individual TAA in cancer was variable but rarely exceeded 20%. With the successive addition of TAAs to a final combination of total of eight antigens, there was a stepwise increase of positive antibody reactions reaching a sensitivity of 63.5% and a specificity of 86.2% in the combined cancer group. In different types of cancer, the ranges of positive and negative likelihood ratio were 4.07–4.76 and 0.39–0.51, respectively, and the ranges of positive and negative predictive values were 74.2–88.7% and 58.8–75.8%, respectively. Agreement rate and Kappa value were 67.1% and 0.51, respectively. These results further support our previous hypothesis that detection of anti-TAAs autoantibodies for diagnosis of certain type of cancer can be enhanced by using a miniarray of several TAAs.

## 1. Introduction

Many studies demonstrated that cancer sera contain antibodies which react with a unique group of autologous cellular antigens generally known as tumor-associated antigens (TAAs) [1, 2]. The types of cellular proteins which induce autoantibody responses are quite varied and include tumor suppressors such as p53 [3] and p16 [4], mRNA-binding proteins such as p62 [5], cell-cycle control proteins such as cyclin B1 [6, 7], and other cancer-related proteins. The immune systems of certain cancer patients are able to sense these aberrant tumor-associated proteins as unknown antigens and have the capability to respond by producing autoantibodies [8, 9]. Although the mechanism underlying the production of such autoantibodies in cancer patients is not completely understood, these autoantibodies can be used as reporters identifying aberrant cellular mechanisms in tumorigenesis and also serve as immunodiagnostic markers for cancer detection [1, 2, 10].

Many investigators have been interested in the use of autoantibodies as serological markers for cancer diagnosis, especially because of the general absence of these autoantibodies in normal individuals and noncancer conditions. Enthusiasm for this approach has been tempered by the low sensitivity. We have observed that this drawback can be overcome by using a panel of carefully selected TAAs and that different types of cancer may require different panels of TAAs to achieve the sensitivity and specificity required to make immunodiagnosis a feasible adjunct to tumor diagnosis [11–15]. This feature is one of the innovative notions we have proposed in our study. For example, a previous study showed that the frequency of antibodies to any individual antigen rarely exceeded 15–20%, but with the successive addition of TAAs to a final combination of total seven antigens, there was stepwise increase in the percentage of positive reactors between 44% and 68% against a combined panel of seven antigens [16]. In addition, breast, lung, and prostate cancers showed separate and distinctive profiles of antibody responses. It is conceivable that tailor-made TAA panels or arrays could be developed for different cancers and that TAA miniarrays might provide another approach to tumor detection and diagnosis.

In the present study, we determine whether a miniarray of multiple TAAs would enhance autoantibody detection and be a useful approach to cancer detection and diagnosis. In addition, this study also carries out evaluation of the diagnostic value of autoantibodies to a panel of multiple TAAs in different types of cancer.

## 2. Materials and Methods

### 2.1. Serum Samples

Sera from 304 patients with different types of cancer (98 lung cancer, 50 hepatocellular carcinoma, 46 colorectal cancer, 41 gastric cancer, and 69 other cancers including 15 bladder cancer, 14 pancreatic cancer, 12 breast cancer, 8 esophageal cancer, 7 ovarian cancer, 7 renal carcinoma, and 6 prostate cancer) and 58 normalhuman sera were obtained from the Department of Clinical laboratory Technology of Dalian Municipal Central Hospital (Liaoning Province, China). All cancer sera were collected at one time of cancer diagnosis when the patients had not yet received treatment with any chemotherapy or radiotherapy; 58 normal human sera were collected from adults during annual health examination in people who had no obvious evidence of malignancy. Due to regulations concerning studies of human subjects, the patient's name and identification number were blinded to investigators. This study was approved by the Institutional Review Boards of Dalian Municipal Central Hospital and collaborating academic institutions.

### 2.2. Recombinant TAAs

All TAAs used in this study, including Imp1, p62, Koc, p53, C-myc, Cyclin B1, Survivin, and p16, were constructed and purified from our previous studies [12, 14]. The reactivities of these selected TAAs were determined with either polyclonal or monoclonal antibodies against the respective proteins.

### 2.3. Enzyme-Linked Immunosorbent Assay (ELISA)

Purified recombinant proteins (Imp1, p62, Koc, p53, C-myc, Cyclin B1, Survivin, and p16) were individually diluted in phosphate buffered saline (PBS) to a final concentration of 0.5 *μ*g/mL, and 200 *μ*L was pipetted into each well to coat onto microtitre plates (Gibico, USA) overnight at 4°. The human serum samples were diluted in serum diluent at 1 : 200, incubated with the antigen-coated wells at 37° for 90 minutes followed by washing with PBS containing 0.05% Tween-20 (PBST) and then incubated with horseradish peroxidase- (HRP-) conjugated goat anti-human IgG (Caltag Laboratory, USA) as a secondary antibody diluted in anti-immunoglobulin diluent at 1 : 4,000 for 90 minutes followed by washing with PBST. The solution of 3,3′,5,5′-TMB (3,3′,5,5′-tetramethyl benzidine, TMB) was used as the detecting agent. The OD of each well was read at 450 nm. Each sample was tested in duplicate. The cut-off value for determining a positive reaction was designated as the mean absorbance of the 58 normal human sera plus 2 standard deviations (mean + 2SD). Because several hundreds of tests with sera were analyzed at different time periods, each run of ELISA included 4 NHS samples and 2 positive control samples. Four NHS samples representing a range of 2SD above and below the mean of the 58 normal human sera were always used in each experiment and the average value of these 4 NHS samples was used in each run to normalize all absorbance values to the standard mean of the entire 58 normal samples. In addition, all positive sera were confirmed with repeat testing, as were some negative sera. The detailed protocol of ELISA has been described previously [5, 16].

### 2.4. Western Blotting

Serum samples that were determined, using ELISA method, to contain autoantibodies were further tested by western blotting to confirm the immunoreactivity to TAAs. In brief, the purified recombinant proteins (Imp1, p62, Koc, p53, c-myc, cyclin B1, survivin, and p16) were electrophoresed by SDS-PAGE and subsequently transferred to a nitrocellulose membrane. After blocking in TBST with 5% nonfat milk for 2 hours at room temperature, the PVDF membrane was incubated for 90 minutes with patient's sera diluted in serum diluent at 1 : 100 and then incubated with HRP-conjugated goat anti-human IgG diluted at 1 : 3000 for 90 minutes followed by washing with TBST solution. The ECL-kit was used to detect immunoreactive bands according to the manufacturer's instructions (Kangwei Biological Technology Company, Beijing, China).

### 2.5. Statistical Analysis

To determine whether the frequency of autoantibodies to eight TAAs in each cohort of patients' sera was significantly higher than that in sera from normal cohort, the data were analyzed using the *χ* ^2^ tests with Yates' correction. Two statistically significant levels (0.05 and 0.01) were used. The comprehensive evaluation of testing result for each anti-TAA antibody including the methods for calculating the sensitivity, specificity, Youden's index, positive and negative likelihood ratios, positive and negative predictive values, agreement rate, and Kappa value was based on the methodology provided in Epidemiology textbook [17].

## 3. Results

### 3.1. Frequency of Autoantibodies to the Miniarray of Eight TAAs

**Figure 1:**
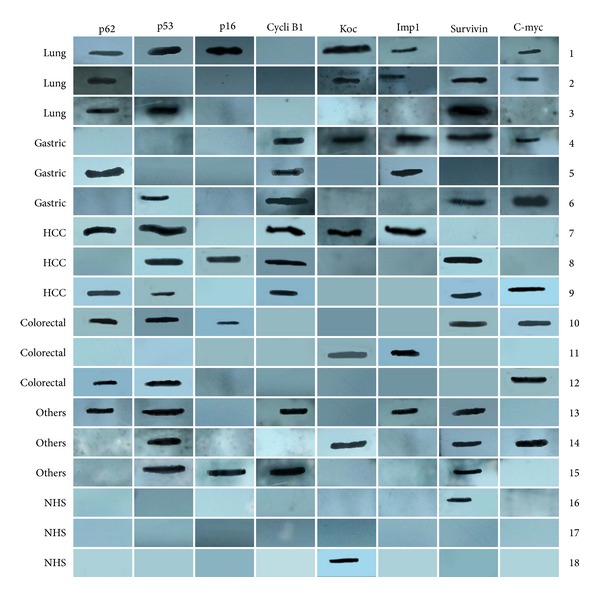
Miniarray of multiple TAAs with representative cancer sera using western blot analysis. Lanes 1–3 are three representative lung cancer sera; lanes 4–6 are three representative gastric cancer sera; lanes 7–9 are three representative hepatocellular carcinoma (HCC) sera; lanes 10–12 are three representative colorectal cancer sera; lanes 13–15 are three representative other cancers sera, showing different antibody profiles with the eight TAAs; and lanes 16–18 are three representative normal human sera (NHS), showing positive reactivity to Koc and survivin but not with other TAAs.

In order to evaluate the diagnostic values of antibodies to multiple TAAs in immunodiagnosis of cancer, eight purified recombinant TAAs were used as coating antigens in ELISA to detect autoantibodies against these eight TAAs in different types of cancer in this study. A positive test for anti-TAAs antibodies was taken as an absorbance reading above the mean + 2SD of the 58 normal human sera. As shown in Table 1, the frequency of autoantibodies to a miniarray of eight TAAs in sera from 304 patients with different types of cancer was variable, ranging between 4.9% and 26.8%. The highest frequency was cyclin B1 (26.8%) in gastric cancer. The cumulative autoantibody frequencies to eight TAAs were 64.3%, 66.0%, 65.2%, 56.1%, and 63.8% in different types of cancer, significantly higher than the frequency in sera from normal individuals (13.8%). The ELISA results were also confirmed by immunoblotting analysis.  Figure 1 shows a miniarray analysis of eight antigens with 18 representative sera using western blot.

Differences in the reactions of different cancers to TAAs were observed and variations in the frequency of anti-TAAs antibodies were observed for any antigen. Positive reaction to Koc was detected in HCC, lung, colorectal, and other cancers but there was no significant difference between gastric cancer and normal individuals. For anti-C-myc antibody, a significant increased frequency was found in lung, colorectal, gastric, and other cancers, but there was no increased frequency in HCC. It is apparent from the data in Table 1 that different profiles of anti-TAAs antibodies could be observed using this array of eight TAAs.

An interesting feature we have observed in this study was that the highest frequencies of anti-TAAs antibody were 26.8% (anti-cyclin B1) in gastric cancer and 25.5% (anti-p53) in lung cancer. As noted previously by many investigators, the likelihood that antibodies would be detected against any individual TAA did not reach the level of high sensitivity, which would be useful as diagnostic biomarker. However, using a miniarray of eight TAAs, the number of anti-TAAs positive reactions increased to 56.1% in gastric cancer and 66.0% in HCC. For a total of 304 cancer patients, the sensitivity of the eight TAA arrays was 63.5% and the specificity was 86.2%. These data indicate that the use of the multiple TAAs can increase the sensitivity of anti-TAAs antibody detection in cancer sera.

### 3.2. Stepwise Increase in Rate of Anti-TAAs Antibody Positivity with Successive Addition of TAAs

Table 2 shows that the sequential addition of antigens to the array resulted in a stepwise increase in the number of positive reactions. With the successive addition of TAAs to a final combination of total eight antigens, this varied from one cancer to another and also from one antigen to another. The addition of CyclinB1 to Koc, p62 and Imp1 in the antigen array increased the number of positive reactions in gastric cancer (24.4–39.0%) and other cancers (26.1–42.0%), but there were lower increases in colorectal cancer (43.5–47.8%). In gastric cancer, the addition of p53 did not further increase the number of positive reactors compared with other types of cancers. These observations suggest that, for certain types of cancer, some antigens may turn out to be more specific while others may not.

### 3.3. Evaluation of the Diagnostic Values of a Panel of Eight TAAs in the Immunodiagnosis of Cancer

The validity of a test method is defined as its ability to distinguish between individuals with a disease and those without the disease. In order to assess the diagnostic value of this approach using a miniarray of multiple TAAs in separating individuals with and without cancer, a group of parameters, such as the YI, sensitivity/specificity, and PPV/NPV, were calculated and are shown in Tables 3 and 4.

Table 3 shows the comprehensive evaluation of antibodies to a panel of eight TAAs. With the successive addition of TAAs to a total of eight antigens, there was a stepwise increase of positive antibody reactions up to 63.5% and there was also a slight decrease of specificity from 100.0% with one TAA to 86.2% with a panel of eight TAAs. It is consistent with the results of two other parameters (PPV/NPV). The PPV/NPV were also variable in different combinations of TAAs. In the panel of eight TAAs, the ranges of the PPV and the NPV in different types of cancer were 74.2–88.7%, and 58.8–75.8%, respectively. Youden's index also increased from 0.049–0.180 with one TAA to 0.423–0.522 with eight TAAs. The ranges of positive and negative likelihood ratios were, respectively, 4.07–4.76 and 0.39–0.51 in different cancer cohort and in total cancer group were 4.60 and 0.42, respectively, which showed that the clinical diagnostic value of parallel assay of eight TAAs was high. Positive and negative predictive values were 74.2–88.7% and 58.8–75.8%, respectively. It indicated that parallel assay of eight TAAs raised the diagnostic accuracy greatly. Agreement rate and Kappa were 72.4–74.0% and 0.44–0.53, respectively, which indicated the observed value of this assay had middle range of coincidence with actual value. These data suggest that using a miniarray of multiple TAAs can increase the clinical diagnostic quality and value in cancer.

## 4. Discussion

Numerous studies indicate that no single marker can completely identify and differentiate the cancer groups from healthy controls. However, a combination of multiple markers may provide a promising way for the early detection of cancer. On the other hand, the multifactorial and multistep nature of the molecular pathogenesis of human cancer can also be considered in the design and interpretation of studies to identify biomarkers useful for the early detection of cancer. Our previous studies showed that combinations of multiple antigen-antibody systems might acquire higher sensitivity for diagnosis of cancer [13, 18]. Wang et al. used a phage display library derived from prostate cancer tissue to develop a phage protein microarray for the analysis of autoantibodies in serum samples from 119 patients with prostate cancer and 138 individuals with no history of prostate cancer [19]. In this study, a 22-phage-peptide detector was constructed for prostate-cancer screening, with 81.6% sensitivity and 88.2% specificity. These studies strongly support the hypothesis that “customized” TAA arrays enhance autoantibody detection in cancer and constitute promising and powerful tools for the immunoserological diagnosis of certain cancer.

In the present study, eight TAAs were used as coating antigens in 304 sera from patients with different types of cancer and 58 sera from normal individuals. With the successive addition of TAAs to a final combination of total eight antigens, the sensitivity in detecting autoantibody in any type of cancer increased from 20% to 26% when one antigen was used, while it ranged from 56% to 66% when eight antigens were used. In the combined cancer group, positive predictive value was 96.0%. It indicated that parallel assay of eight TAAs raised the diagnostic quality greatly. In addition, positive likelihood ratio was 4.60, which showed that the clinical diagnostic value of parallel assay of eight TAAs was high, and Kappa value was 0.51, which indicated the observed value of this assay had middle range of coincidence with actual value.

Our aim is to increase the sensitivity and specificity of anti-TAA antibodies as diagnostic markers in cancer detection by expanding the TAA array, including TAAs which may be more selectively associated with one specific type of cancer and not with others. For future studies, we propose that certain selected antibody-antigen systems may be unique to one type of cancer and others may not. Optimum candidates for inclusion in a miniarray of multiple TAAs and the specific panels of TAAs should be developed for different cancers. We stress the notion that panels of “customized” TAAs should be used for different types of cancer and that these customized panels should be rigorously tested for sensitivity and specificity not only against other cancers but also against other disease conditions. For instance, in the case of lung cancer, the nature of precondition would be heavy cigarette smokers. It has been well known that cigarette smoking is the major cause of lung cancer and is currently estimated to cause 85% of all lung cancer deaths. In the case of HCC, the natural conditions would be chronic hepatitis and liver cirrhosis. Cancer-associated antigen panels might conceivably be used for early detection of tumors in high-risk individuals. Anti-p53 antibodies were detected in two heavy smokers before clinical detection of lung cancer, and, in one patient, early treatment resulted in good response, which correlated with total disappearance of p53 antibodies [20, 21]. The basis for the notion of the customized panels of TTAs is to identify a specific panel of TAAs for one type of cancer and compare this with antigen panels associated with the natural conditions or high-risk individuals.

In conclusion, this study further supports our previous hypothesis that a customized miniarray of multiple carefully selected TAAs might acquire higher sensitivity for the diagnosis of cancer. TAA arrays provide promising and powerful tools for enhancing cancer detection, but their utility in a clinical setting is currently still in its infancy. Before TAA arrays could be widely implemented in screening programs for cancer diagnosis or as tools for monitoring cancer progression and guiding therapeutic interventions, it would be important to maximize their sensitivity and specificity by defining systematically the optimal combination of TAAs.

## 5. Conclusion

This study demonstrates that detection of autoantibodies for diagnosis of certain types of cancer can be enhanced by using a miniarray of several TAAs as target antigens. These results also indicated that the design of unique TAA panels for different cancers would help to determine whether using a miniarray of multiple TAAs is a clinically useful noninvasive approach in cancer detection and diagnosis.

## Figures and Tables

**Table 1:** Frequency of antibodies to eight TAAs.

Autoantibodies to	Number (%) of autoantibodies in different types of cancers						
	Lung (98)	HCC (50)	Colorectal (46)	Gastric (41)	Others (69)	Total (304)	NHS (58)
Koc	9 (9.2)^a^	9 (18.0)^b^	7 (15.2)^b^	2 (4.9)	8 (11.6)^a^	35 (11.5)^b^	0
p62	15 (15.3)^b^	10 (20.0)^b^	10 (21.7)^b^	4 (9.8)	9 (13.0)^a^	48 (15.8)^b^	1 (1.7)
Imp1	17 (17.3)^b^	8 (16.0)^b^	10 (21.7)^b^	6 (14.6)^b^	9 (13.0)^a^	50 (16.4)^b^	0
Cyclin B1	17 (17.3)^b^	10 (20.0)^b^	8 (17.4)^a^	11 (26.8)^b^	12 (17.4)^b^	58 (19.1)^b^	1 (1.7)
p16	18 (18.4)^b^	3 (6.0)	9 (19.6)^a^	4 (9.8)	13 (18.8)^b^	47 (15.5)^a^	2 (3.4)
Survivin	19 (19.4)^b^	6 (12.0)	7 (15.2)^a^	6 (14.6)^a^	10 (14.5)^a^	48 (15.5)^b^	1 (1.7)
C-myc	24 (24.5)^b^	7 (14.0)	10 (21.7)^b^	7 (17.1)^a^	17 (24.6)^b^	65 (21.4)^b^	2 (3.4)
p53	25 (25.5)^b^	10 (20.0)^a^	11 (23.9)^b^	5 (12.2)	14 (20.3)^b^	65 (21.4)^b^	2 (3.4)
Cumulative to eight antigens	63 (64.3)^b^	33 (66.0)^b^	30 (65.2)^b^	23 (56.1)^b^	44 (63.8)^b^	193 (63.5)^b^	8 (13.8)

Cutoff value: mean +2 SD of NHS; *P* values to NHS were calculated to be <0.05 (^a^) or 0.01 (^b^).

HCC: hepatocellular carcinoma; NHS: normal human sera.

**Table 2:** Sequential addition of antigens to the miniarray of eight TAAs.

Number of different TAA panels	Number and percentage of autoantibodies in different types of cancers						
	Lung (98)	HCC (50)	Colorectal (46)	Gastric (41)	Others (69)	Total (304)	NHS (58)
(1) Koc	9 (9.2)^a^	9 (18.0)^b^	7 (15.2)^b^	2 (4.9)	8 (11.6)^a^	35 (11.5)^b^	0 (0)
(2) Koc and p62	20 (20.4)^b^	17 (34.0)^b^	15 (32.6)^b^	6 (14.6)^a^	15 (21.7)^b^	73 (24.0)^b^	1 (1.7)
(3) Koc, p62, and Imp1	31 (31.6)^b^	22 (44.0)^b^	20 (43.5)^b^	10 (24.4)^b^	18 (26.1)^b^	101 (33.2)^b^	1 (1.7)
(4) Koc, p62, Imp1, and cyclin B1	41 (41.8)^b^	26 (52.0)^b^	22 (47.8)^b^	16 (39.0)^b^	29 (42.0)^b^	134 (44.1)^b^	2 (3.4)
(5) Koc, p62, Imp1, cyclin B1, and p16	47 (48.0)^b^	27 (54.0)^b^	24 (52.2)^b^	19 (46.3)^b^	36 (52.2)^b^	153 (50.3)^b^	4 (6.9)
(6) Koc, p62, Imp1, cyclin B1, p16, and survivin	54 (55.1)^b^	29 (58.0)^b^	25 (54.3)^b^	21 (51.2)^b^	38 (55.1)^b^	167 (55.0)^b^	5 (8.6)
(7) Koc, p62, Imp1, cyclin B1, p16, survivin, and C-myc	58 (59.2)^b^	32 (64.0)^b^	28 (60.9)^b^	23 (56.1)^b^	42 (60.9)^b^	183 (60.2)^b^	7 (12.1)
(8) Koc, p62, Imp1, cyclin B1, p16, survivin, C-myc, and p53	63 (64.3)^b^	33 (66.0)^b^	30 (65.2)^b^	23 (56.1)^b^	44 (63.8)^b^	193 (63.5)^b^	8 (13.8)

All *P* values relative to NHS were calculated to be <0.05 (^a^) or 0.01 (^b^); HCC: hepatocellular carcinoma; NHS: normal human sera.

**Table 3:** Evaluation of diagnostic values of different TAA panels in the diagnosis of cancer.

		Number of different TAA panels^a^							
		1	2	3	4	5	6	7	8
NHS	Positive % (number)	0 (0)	1.7 (1)	1.7 (1)	3.4 (2)	6.9 (4)	8.6 (5)	12.1 (7)	13.8 (8)

Lung (98)	Positive % (number)	9.2 (9)	20.4 (20)	31.6 (31)	41.8 (41)	48.0 (47)	55.1 (54)	59.2 (58)	64.3 (63)
	Se/Sp	9.2/100.0	20.4/98.3	31.6/98.3	41.8/96.6	48.0/93.1	55.1/91.4	59.2/87.9	64.3/86.2
	YI	0.092	0.187	0.299	0.384	0.411	0.465	0.471	0.505
	PPV/NPV	100.0/39.5	95.2/42.2	96.9/46.0	95.3/49.6	92.2/51.4	91.5/54.6	89.2/56.0	88.7/58.8

HCC (50)	Positive % (number)	18.0 (9)	34.0 (17)	44.0 (22)	52.0 (26)	54.0 (27)	58.0 (29)	64.0 (32)	66.0 (33)
	Se/Sp	18.0/100.0	34.0/98.3	44.0/98.3	52.0/96.6	54.0/93.1	58.0/91.4	64.0/87.9	66.0/86.2
	YI	0.180	0.323	0.423	0.486	0.471	0.494	0.520	0.522
	PPV/NPV	100.0/58.6	94.4/63.3	95.7/67.1	92.9/70.0	87.1/70.1	85.3/71.6	82.1/73.9	80.5/74.6

Colorectal (46)	Positive % (number)	15.2 (7)	32.6 (15)	43.5 (20)	47.8 (22)	52.2 (24)	54.3 (25)	60.9 (28)	65.2 (30)
	Se/Sp	15.2/100.0	32.6/98.3	43.5/98.3	47.8/96.6	52.2/93.1	54.3/91.4	60.9/87.9	65.2/86.2
	YI	0.152	0.309	0.418	0.444	0.453	0.457	0.488	0.514
	PPV/NPV	100.0/59.8	93.8/63.3	95.2/68.7	91.7/70.0	85.7/71.1	83.3/71.6	80.0/73.9	78.9/75.8

Gastric (41)	Positive % (number)	4.9 (2)	14.6 (6)	24.4 (10)	39.0 (16)	46.3 (19)	51.2 (21)	56.1 (23)	56.1 (23)
	Se/Sp	4.9/100.0	14.6/98.3	24.4/98.3	39.0/96.6	46.3/93.1	51.2/91.4	56.1/87.9	56.1/86.2
	YI	0.049	0.129	0.227	0.356	0.394	0.426	0.440	0.423
	PPV/NPV	100.0/59.8	85.7/62.0	90.9/64.8	88.9/69.1	82.6/71.1	80.8/72.6	76.7/73.9	74.2/73.5

Others (69)	Positive % (number)	11.6 (8)	21.7 (15)	26.1 (18)	42.0 (29)	52.2 (36)	55.1 (38)	60.9 (42)	63.8 (44)
	Se/Sp	11.6/100.0	21.7/98.3	26.1/98.3	42.0/96.6	52.2/93.1	55.1/91.4	60.9/87.9	63.8/86.2
	YI	0.116	0.200	0.244	0.386	0.453	0.465	0.488	0.500
	PPV/NPV	100.0/48.7	93.8/51.4	94.7/52.8	93.5/58.3	90.0/62.1	88.4/63.1	85.7/65.4	84.6/66.7

Total (304)	Positive % (number)	11.5 (35)	24.0 (73)	33.2 (101)	44.1 (134)	50.3 (153)	55.0 (167)	60.2 (183)	63.5 (193)
	Se/Sp	11.5/100.0	24.0/98.3	33.2/98.3	44.1/96.6	50.3/93.1	55.0/91.4	60.2/87.9	63.5/86.2
	YI	0.115	0.223	0.315	0.407	0.434	0.464	0.481	0.497
	PPV/NPV	100.0/17.7	98.6/19.8	99.0/21.9	98.5/24.8	97.5/26.3	97.1/27.9	96.3/29.7	96.0/31.1

^a^Number of different TAA panels, corresponding to TAA panels shown in Table 2.

Se: sensitivity; Sp: specificity; YI: Youden's index; PPV: positive predictive value; NPV: negative predictive value.

**Table 4:** Summary of diagnostic value of antibodies to a panel of eight TAAs.

	Lung	HCC	Colorectal	Gastric	Others	Total	NHS
Any TAA positive	63 (A1)	33 (A2)	30 (A3)	23 (A4)	44 (A5)	193 (A6)	8 (B)
All TAA negative	35 (C1)	17 (C2)	16 (C3)	18 (C4)	25 (C5)	111 (C6)	50 (D)
Youden's index	0.505	0.522	0.514	0.423	0.500	0.497	—
+Likelihood ratio	4.66	4.76	4.72	4.07	4.62	4.60	—
−Likelihood ratio	0.41	0.39	0.45	0.51	0.42	0.42	—
+Predictive value	88.7	80.5	78.9	74.2	84.6	96.0	—
−Predictive value	58.8	74.6	75.8	73.5	66.7	31.1	—
Agreement rate	72.4	76.9	76.9	73.7	74.0	67.1	—
Kappa value	0.46	0.53	0.52	0.44	0.49	0.51	—

Note: Calculation Formulas used in Table 4 were as follows.

Youden's index = sensitivity + specificity − 1.

Positive (+) likelihood ratio = sensitivity/(1 – specificity).

Negative (−) likelihood ratio = (1 – sensitivity)/specificity.

Positive (+) predictive value = A/(A + B) × 100%.

Negative (−) predictive value = D/(C + D) × 100%.

Agreement rate = (A + D)/(A + B + C + D) × 100%.

Kappa value = [N(A + D)−(R_1_C_1_ + R_2_C_2_)]/[N_2_ − (R_1_C_1_ + R_2_C_2_)].
